# Bacterially Produced Recombinant Influenza Vaccines Based on Virus-Like Particles

**DOI:** 10.1371/journal.pone.0078947

**Published:** 2013-11-18

**Authors:** Andrea Jegerlehner, Franziska Zabel, Alice Langer, Klaus Dietmeier, Gary T. Jennings, Philippe Saudan, Martin F. Bachmann

**Affiliations:** Immunodrugs Department, Cytos Biotechnology AG, Schlieren, Zurich, Switzerland; University of Georgia, United States of America

## Abstract

Although current influenza vaccines are effective in general, there is an urgent need for the development of new technologies to improve vaccine production timelines, capacities and immunogenicity. Herein, we describe the development of an influenza vaccine technology which enables recombinant production of highly efficient influenza vaccines in bacterial expression systems. The globular head domain of influenza hemagglutinin, comprising most of the protein's neutralizing epitopes, was expressed in *E. coli* and covalently conjugated to bacteriophage-derived virus-like particles produced independently in *E.coli.* Conjugate influenza vaccines produced this way were used to immunize mice and found to elicit immune sera with high antibody titers specific for the native influenza hemagglutinin protein and high hemagglutination-inhibition titers. Moreover vaccination with these vaccines induced full protection against lethal challenges with homologous and highly drifted influenza strains.

## Introduction

Influenza virus is one of most important infectious agents having killed more people in the 20th century than any other virus. Vaccination is a potent and cost-effective measure against seasonal and pandemic outbreaks of influenza. The efficacy of current vaccines is based on induction of neutralizing antibodies against the main surface glycoprotein of influenza, hemagglutinin (HA). The HA protein has important functions in sialic acid receptor binding and membrane fusion during virus infection. Based on antigenic classification, 16 antigenic subtypes of HA have been described [1], [2]. The annual trivalent inactivated influenza vaccines are composed of two currently circulating influenza A strains (H1N1 and H3N2), together with an influenza B strain [3].

The vast majority of current flu vaccines are produced using egg-based manufacturing systems which while effective, are hampered by limited capacity and flexibility [4]. Recombinant production of influenza HA could therefore be an attractive alternative [5]. However, to date this approach has two disadvantages, namely low productivity and low immunogenicity of recombinant HA [6]. To address these issues we set out to develop influenza vaccines fully produced in bacteria that exhibit the immunogenicity of inactivated viruses rather than subunit vaccines. In order to improve expression of HA, which is notoriously difficult in eukaryotic cells [7] [8] [9], we rationally designed constructs containing the globular domain of HA for expression in *E. coli* [10], [11], [12]. The globular domain of the HA protein is the most membrane distal part of the protein and the target of most virus neutralizing antibodies [13], [14]. Hence, this domain is ideal for the induction of neutralizing antibodies against influenza virus. We therefore decided to use this domain and to conjugate it to bacteriophage Qβ virus like particles (VLP), which we have previously developed as a carrier for vaccines. With this technology, antigens of choice are rendered highly immunogenic by covalent coupling of them to the surface of the RNA-loaded VLPs [15] [16] [17], [18].

Here we demonstrate that HA globular domains expressed in bacteria, bound sialic acids and, upon coupling to Qβ, induced hemagglutination of red blood cells with comparable efficiency to influenza viral particles. Upon immunization, mice mounted strong HA-specific IgG responses which were hemagglutination inhibiting (HI). Immunization of mice resulted in protection against lethal infection with not only the homologous but also highly drifted viral strains. Hence, the herein described technology may be suited to produce large amounts of effective influenza vaccines with time-lines that could enable vaccination campaigns before rather than after the next pandemic.

## Materials and Methods

### Production of Qβ VLPs

Qβ VLPs were produced as follows: Capsids of the RNA-phage Qβ were expressed using the expression vector pQβ 10. *E. coli* lysates containing the expressed coat protein were cleared by centrifugation. After centrifugation proteins were fractionated by ammonium sulfate precipitation. The precipitated capsids were resuspended in gel filtration buffer containing 20 mM Tris-HCl pH 7.8, 5 mM EDTA and 150 mM NaCl, and purified over a Sepharose CL-4B column (Amersham). Eluted capsids were precipitated with PEG-6000 at 13.3% saturation and re-purified on a Sepharose CL-4B column. Capsids present in the peak fractions were precipitated with ammonium sulfate at 60% saturation. Sedimented VLPs were resuspended in gel filtration buffer and loaded onto a Sepharose CL-6B column (Amersham). Fractions containing the Qβ VLPs were pooled, concentrated by ammonium sulfate precipitation and dialyzed against 20 mM HEPES, 150 mM NaCl, pH 7.4.

### Cloning, expression, purification and refolding of influenza gH proteins

Globular domain fragments of the ectodomain of HA (gH) of mouse adapted influenza A/PR/8/34 (H1N1) virus (prototype gH fragments) were designed based on the protein structure (PDB 1RVX) of prototype human (1934-human) H1 influenza virus A/Puerto Rico/8/34 HA [19]. Based on aa sequence alignment of mouse adapted A/PR/8/34 with the prototype human (1934-human) H1 influenza virus A/Puerto Rico/8/34 HA the nucleotide sequence encoding amino acids 36–311 (HA1) flanked by a NdeI restriction site at the N-terminus and by a XhoI restriction site at the C-terminus was optimized for expression in *E. coli* and produced by gene synthesis (Geneart, Regensburg, Germany). The optimize nucleotide sequence was digested with NdeI and XhoI and cloned into NdeI-XhoI restriction sites of a modified version of pET-42a (+) leading to the addition of the sequence LEHHHHHHGGC at the C-terminus called pET-42T (+) resulting in plasmid pET42T_gH_PR8_36_311. This sequence contains a His-tag followed by a GGC linker used for coupling of the protein to Qβ VLPs. This vector was used to generate different shorter fragments by PCR. These plasmids encode fusion proteins consisting of an N-terminus composed of the aa sequences aa36-311, aa40-311, aa52-277, aa49-277, aa49-271, aa52-271, aa72-277, aa82-277, aa87-261, aa87-263 and aa90-263 of the ectodomain of mouse adapted influenza virus A/PR/8/34 genetically fused to the Nterminus of aa sequence LEHHHHHHGGC at the C-terminus. The resulting proteins were named gH_A1, gH_A2, gH_B1, gH_B2, gH_B3, gH_B4, gH_C1, gH_C2, gH_C3, gH_C4, gH_C5 respectively.

The gH_A1 H1 prototype fragment was structurally aligned to the structure of an influenza HA of the H3 subtype (pdb 1E08) [20] to the structure of an influenza HA of H5 subtype (pdb 2 FK0) [21] and human influenza B virus (pdb 3BT6) [22] to design influenza A H3 prototype, influenza A H5 prototype HA fragments and influenza B prototype HA fragments with similar structures as the influenza gH_A1 H1 HA prototype fragments. Influenza A H1, H3 and H5 and influenza B fragments of influenza viruses from which we wanted to generate gH_A1 globular domain proteins were designed by aa alignment with the prototype HA fragments of the corresponding subtypes of influenza A virus or influenza B virus strains. Cloning into the pET42T vector was performed as described for gH_A1_PR8.

For expression, *Escherichia coli* BL21 cells harboring either plasmid were grown at 37°C to an OD at 600 nm of 1.0 and then induced by addition of IPTG at a concentration of 1 mM. Bacteria were grown for 4 more hours at 37°C, harvested by centrifugation and resuspended in 5 ml lysis buffer (50 mM Na_2_HPO_4_, 300 mM NaCl, 10 mM Imidazole, pH 8.0) per gram wet weight and cells were lysed by 30 min incubation with 1 mg/ml lysozyme. Cells were then disrupted by sonication and cellular DNA was digested by 15 min incubation on ice with 5 μg/ml DNAse I. Inclusion bodies (IB) were harvested by centrifugation (10′000× g, 4°C, 30 min), purified using B-PER I reagent (Pierce) and solubilized in IB solubilisation buffer (8 M urea, 50 mM Tris-Cl pH 8.0, 50 mM Dithiothreitol) to a concentration of 0.5 mg/ml. Refolding of proteins was performed by dialysis against refolding buffer 2 (2 M urea, 50 mM NaH_2_PO_4_, 0.5 M Arginine, 10% Glycerole (v/v), 5 mM Glutathion reduced, 0.5 mM Glutathion oxidized, pH 8.5), followed by dialysis against refolding buffer 3 (50 mM NaH_2_PO_4_, 0.5 M Arginine, 10% Glycerole (v/v), 5 mM Glutathion reduced, 0.5 mM Glutathion oxidized, pH 8.5), followed by dialysis against refolding buffer 4 (20 mM Sodium-Phosphate, 10% Glycerole (v/v), pH 7.2. Refolded proteins were stored at −80°C until further use.

### Conjugation of gH proteins to Qβ VLPs

Coupling of gH proteins to Qβ VLPs was performed and analyzed as described before [15]. Qβ VLPs (in HEPES, pH 7.2) were reacted with a 10 fold molar excess compared to Qβ VLP subunits (VLPs are composed of 180 subunits of 14 kD each) of the heterobifunctional crosslinker succinimidyl-6-(β -maleimidopropionamido)-hexanoate (SMPH) (PIERCE, Rockford, USA). Derivatized Qβ was mixed with a 1.5 fold molar excess compared to Qβ subunits of the following proteins to produce conjugate vaccines: gH_A1 (30 kD), gH_A2 (30 kD), gH_B1 (25 kD), gH_B2 (25 kD), gH_B3 (24 kD), gH_B4 (24 kD), gH_C1 (23 kD), gH_C2 (21 kD), gH_C3 (19 kD), gH_C4 (19 kD) of influenza A/PR/8/34 as well as with gH_AC0409.

Non coupled proteins were removed by size exclusion chromatography using a Sepharose CL4B column.

Coupled products were analyzed on a 4–12% Bis-Tris-polyacrylamide gel under reducing conditions. Several bands of increased molecular weight with respect to Qβ monomer were visible, clearly demonstrating the successful cross-linking of all the globular domain fragments. LPS activity of the Qβ -gH conjugate vaccines as well of the gH proteins alone were measured by LAL assay. The concentrations measured were usually smaller than 1 EU/100 µg.

### Production of the ectodomain of Influenza hemagglutinin of A/PR/8/34 (ecHA_PR8)

Based on H3 numbering, cDNA corresponding to residues 11–329 (HA1) and 1–176 (HA2) of the ectodomain of HA0 of influenza virus A/PR/8/34 codon optimized for expression in sf9 insect cells followed by a trimerizing sequence from the bacteriophage T4 fibritin and a 6× His Tag for purification was cloned into the baculovirus transfer vector pFastBac1 to allow for efficient secretion of recombinant protein. Transfection and virus amplification were carried out according to the baculovirus expression system manual (Invitrogen). For expression, suspension cultures of insect Hi5 cells were cultured in Express Hi5 medium (Invitrogen) and infected at a multiplicity of infection (MOI) of 5 for 3 days. The ecHA_PR8 protein was purified from the supernatant by metal affinity chromatography using Ni-NTA resin (Qiagen). Fractions containing ecHA_PR8 protein were pooled and dialyzed against PBS and purified protein was stored at −80°C until further use.

### Mice

BALB/c mice were purchased from Harlan. All animals were kept under specific pathogen-free conditions at BioSupport AG and were used at 6 weeks of age. Experiments were conducted in accordance with protocols approved by the Swiss Federal Veterinary Office. Mouse body temperature was determined by measuring the rectal temperature of the mice using an electronic thermometer.

### ELISA

ELISA plates (96 well MAXIsorb, NUNC) were coated with recombinant influenza HA (rH) (Protein Sciences) at a concentration of 1 µg/ml in PBS, washed with PBS/0.05% and blocked with 2% BSA/PBS. Serum of individual mice was added, serial dilutions performed. Anti-rH antibodies were detected with HRPO-labeled goat anti-mouse IgG antibodies (Jackson Immuno Research Laboratories). The color reaction was developed with a 0.4 mg/ml solution of 1, 2-ortho-phenylenediamine dihydrochloride (OPD) and stopped with a 5% H_2_SO_4_ solution in H_2_O. Plates were read at 450 nm on an ELISA reader (Biorad Benchmark). Antibody titers are defined as the reciprocals of the dilution needed to achieve 50 percent of the signal measured at saturation (OD50 titers).

### Fetuin binding assay

ELISA plates (96 well MAXIsorb, NUNC) were coated with refolded gH at a concentration of 10 µg/ml in PBS, washed with PBS/0.05% Tween and blocked for 2 hours at 37°C with 2% BSA/PBS. Serial dilutions of fetuin, biotinylated using EZ-Link-Sulfo-NHS-LC Kit (Pierce), at a concentration of 0.01 mg/ml in the first well, were performed and plates were incubated for 2 h at room temperature. Plates were then washed with PBS/0.05% Tween. Biotinylated fetuin bound to the native proteins was detected with HRPO-labeled steptavidin. The color reaction was developed with a 0.4 mg/ml solution of 1, 2-ortho-phenylenediamine dihydrochloride (OPD) and stopped with a 5% H_2_SO_4_ solution in H_2_O. Plates were read at 450 nm on an ELISA reader (Biorad Benchmark). EC50 values were determined as concentrations of fetuin at which half maximal OD was reached.

### Hemagglutination-Inhibition assay

Hemagglutination-Inhibition assay was performed according to the WHO manual (WHO manual on animal influenza diagnosis and surveillance).

### Influenza virus infection of mice

The following influenza A viruses were used in the different studies: A/PR/8/34 (H1N1), A/FM/1/47 (H1N1), A/Aichi/2/68 (X31) (H3N2) and A/WSN/33 (H1N1). To determine the lethal dose of each virus, mice were administered serial dilutions of virus (2×50 μl) via the nose under light anesthesia with isoflurane. Body weight and body temperature of infected mice were monitored for at least 20 days after infection. Mice, which had lost more than 30% of their initial body weight or had a body temperature equal to or lower than 30°C were euthanized. LD50 titres were calculated for each virus strain according to the method of Reed and Munch. To determine the efficacy of the different vaccines, mice were immunized with the indicated compounds and challenged with a lethal dose of homologous or heterologous influenza virus (4× LD50 or 10× LD50) as indicated in the respective examples and monitored as described above. Mice that had lost more than 30% of their initial body weight or had a body temperature equal to or lower than 30°C were euthanized. The % surviving animals 20 days post infection (p.i.) for each treatment group is indicated in the respective examples.

### Hemagglutination assay

Qβ-gH_A1 solutions were serially diluted in PBS and mixed with 50 μl of 1% chicken RBCs in 96 well plates. The plates were mixed by agitation, covered, and the RBCs were allowed to settle for 1 h at room temperature. The minimal concentration of Qβ-gH_A1 which still was able to agglutinate the chicken RBCs was determined and was 55 pg/ml.

## Results

### Rational design of HA1 fragments

**Figure 1:**
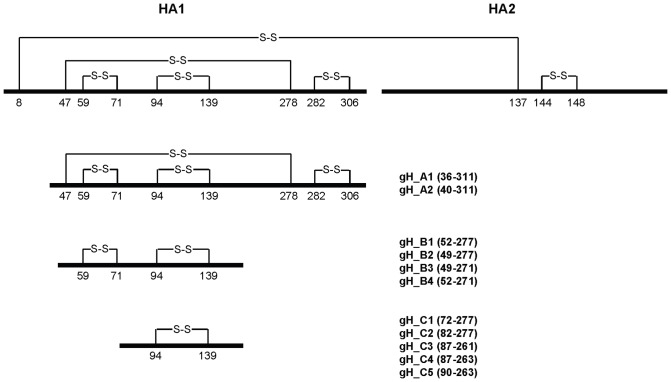
Design and evaluation of globular head fragments. A Schematic of the linear structure of the full-length influenza HA protein and of the rationally designed HA1 globular head fragments.

Determination of the structure of the hemagglutinin-esterase-fusion (HEF) protein of influenza C virus and its comparison with the structure of the HA protein of influenza A and B virus revealed that the HEF protein can be segregated into three structurally distinct domains. These domains fulfill three distinct functions, which are receptor binding, membrane fusion and receptor cleavage by an esterase-activity [23]. These domains therefore might be independent folding units. The HA proteins of influenza A and B contain only a fragment of the functional esterase domain (vestigial esterase domain) and hence only functions in receptor binding and membrane fusion. The receptor binding domain and the vestigial esterase domain are a fragment of HA1 which structurally form a globular domain, which may itself be an independent folding entity which protrudes from the fusion domain. Full length HA1 [12] as well as fragments of HA1 [10], [11] have been recombinantly expressed as inclusion bodies in *E. coli* and refolded *in vitro*. Since the antigenic sites of HA1 are conformational epitopes, the challenge of this approach is to obtain correctly-folded proteins. We have reevaluated some of these fragments and based on the structure of the HA protein (structure: 1RVX) of influenza A/PuertoRico/8/34 [19] we rationally designed eleven fragments of HA1 (gH proteins) which contained four (group A), two (group B) or one (group C) disulfide bridge (s) and which comprise intact secondary structure elements (Fig. 1). All constructs had an additional C-terminal linker with a free Cys for subsequent coupling to Qβ-VLPs.

### Expression and refolding of gH proteins

All gH-fragments were expressed as inclusion bodies, washed, solubilized and refolded by dialysis in phosphate buffer in the presence of a redox-shuffling system. Highest yields of refolded proteins were obtained with constructs containing four disulfide bridges (results not shown). Correct refolding of the proteins was assessed by determining the capacity of the proteins to bind to sialic acid on the fetuin glycoprotein. Refolded proteins were coated on ELISA plates and binding of biotinylated fetuin was detected by HRPO labeled streptavidin. As can be seen in Table 1, the gH_A fragments which contain 4 disulfide bridges exhibited stronger binding than the gH_B fragments which contain 2 disulfide bridges. The lowest binding was observed with the gH_C fragments which contain only one disulfide bridge.

**Table 1:** Receptor binding and Immunogenicity of gH proteins.

Antigen	Binding to Fetuin [ec50, nM]	Anti-rH ELISA titer [OD50]
gH_A1	0.14	18′530
gH_A2	0.06	9′582
gH_B1	1.3	5′589
gH_B2	5.3	1′663
gH_B3	0.7	892
gH_B4	5.0	431
gH_C1	7	170
gH_C2	12	822
gH_C3	14	343
gH_C4	8	20
gH_C5	14	36

ELISA plates were coated with gH proteins and titrating amounts of fetuin were added. EC50 values were determined as concentrations of fetuin at which half maximal OD was reached. BALB/c mice were immunized subcutaneously once with 50 µg of gH proteins in Alum and IgG antibody titers against native trimeric full-length HA protein were determined three weeks later by ELISA.

### Immunogenicity of gH proteins

BALB/c mice were immunized subcutaneously once with 50 μg of gH proteins in Alum and IgG antibody titers against native trimeric full-length HA were determined three weeks later by ELISA. Table 1 shows that the highest antibody titers were induced with globular domain constructs containing 4 disulfide bridges. Comparing ELISA titers versus fetuin-affinity identified 2 populations of domains, characterized by high affinity and high antibody titers (gH_A and B) versus low affinity and low antibody titers (gH_C) (Table 1). The latter domains were thus not pursued further since they appeared to be principally misfolded.

### Immunogenicity and *in vivo* efficacy of Qβ-gH vaccines

Next we wanted to assess whether conjugation of the globular domain HA proteins to Qβ VLPs could enhance their immunogenicity. To this end, the globular domain HA proteins were conjugated to Qβ VLPs using the heterobifunctional cross-linker SMPH. All conjugate vaccines were soluble with the exception of Qβ -gH_B4 which was thus excluded from further experimentation.

**Figure 2:**
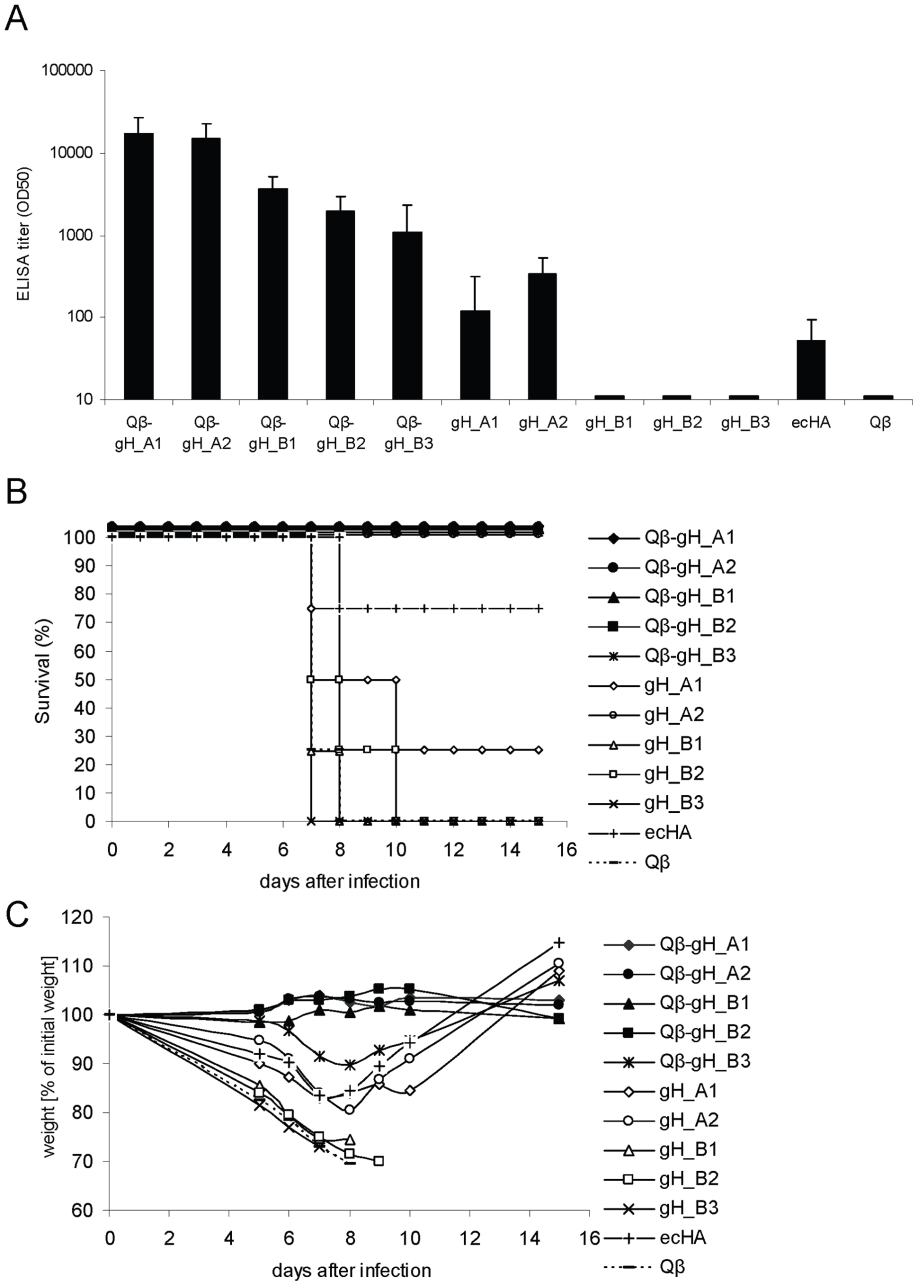
Immunogenicity and *in vivo* efficacy of Qβ -gH vaccines of influenza A/PR/8/34. **A** Groups of six-weeks old female BALB/c mice (n = 4) were immunized subcutaneously once with 15 μg of Qβ -gH conjugate vaccines, 15 μg gH proteins alone, 15 μg full-length trimeric ectodomain of HA or 15 μg Qβ in the absence of adjuvants. IgG antibody titers specific for native HA protein of A/PR/8/34 were measured at day 21 after immunization. ELISA titers are indicated as IgG titer at which half-maximal OD was reached (OD50). **B,C** On day 27, mice were challenged i.n. with 10× LD50 of influenza virus A/PR/8/34. Survival **B** and weight loss **C** of individual mice were monitored for 16 days post challenge. The following survival rates were achieved: Qβ -gH_A1 (100%), Qβ -gH_A2 (100%), Qβ -gH_B1 (100%), Qβ -gH_B2 (100%), Qβ -gH_B3 (100%), gH_A1 (25%), gH_A2 (100%), gH_B1 (0%), gH_B2 (0%), gH_B3 (0%), ecHA (75%), Qβ (0%).

Groups of six-week old female BALB/c mice (n = 4) were immunized once with either 15 μg of the conjugate vaccines or 15 μg of the globular domain alone or, as a comparator, 15 μg of the full-length ecHA or with 15 μg Qβ alone. Please note that 15 µg of conjugate vaccines correspond to total amounts of conjugate vaccine and not amounts of globular domain protein contained in the vaccine. All mice immunized with conjugate vaccine showed significantly higher IgG antibody titers (about 100-fold) against native HA protein than mice immunized with globular domain proteins alone or ecHA three weeks after immunization (Figure 2A). Consistent with results obtained with the free domains, highest titers were reached with Qβ -gH_A1 and Qβ -gH_A2 conjugate vaccines.

Next we explored the protective potential of these antibodies in an *in vivo* challenge model, which is routinely used to assess preclinical efficacy of influenza vaccines. Immunized mice were infected intranasally (i.n.) with a lethal dose (10× LD50) of mouse adapted (m.a.) influenza A/PR/8/34 strain and animals were monitored for morbidity and mortality. As shown in Figure 2B, control mice immunized with Qβ VLP alone or globular domain proteins containing 2 disulfide bridges quickly succumbed to infection, whereas mice immunized with free globular domain proteins containing 4 disulfide bridges and with the native ecHA were partially protected from the influenza virus infection. Although antibody titers of mice immunized with ecHA were slightly lower than of mice immunized with gH_A1, ecHA immunized mice surprisingly showed a survival rate of 75% compared to 25% of gH_A1 immunized mice. In contrast, all animals immunized with conjugate vaccines survived the lethal challenge and, with the exception of Qβ -gH_B3 immunized animals, showed no signs of morbidity (Figure 2C). There was a strong correlation between IgG antibody titers measured by ELISA on full-length HA and protection against influenza virus infection. The most promising candidates were the conjugate vaccines of globular domain proteins with 4 disulfide bridges, namely Qβ -gH_A1 and Q-β gH_A2.

### Titration of Qβ -gH_A1 and Qβ -gH_A2 vaccines

**Figure 3:**
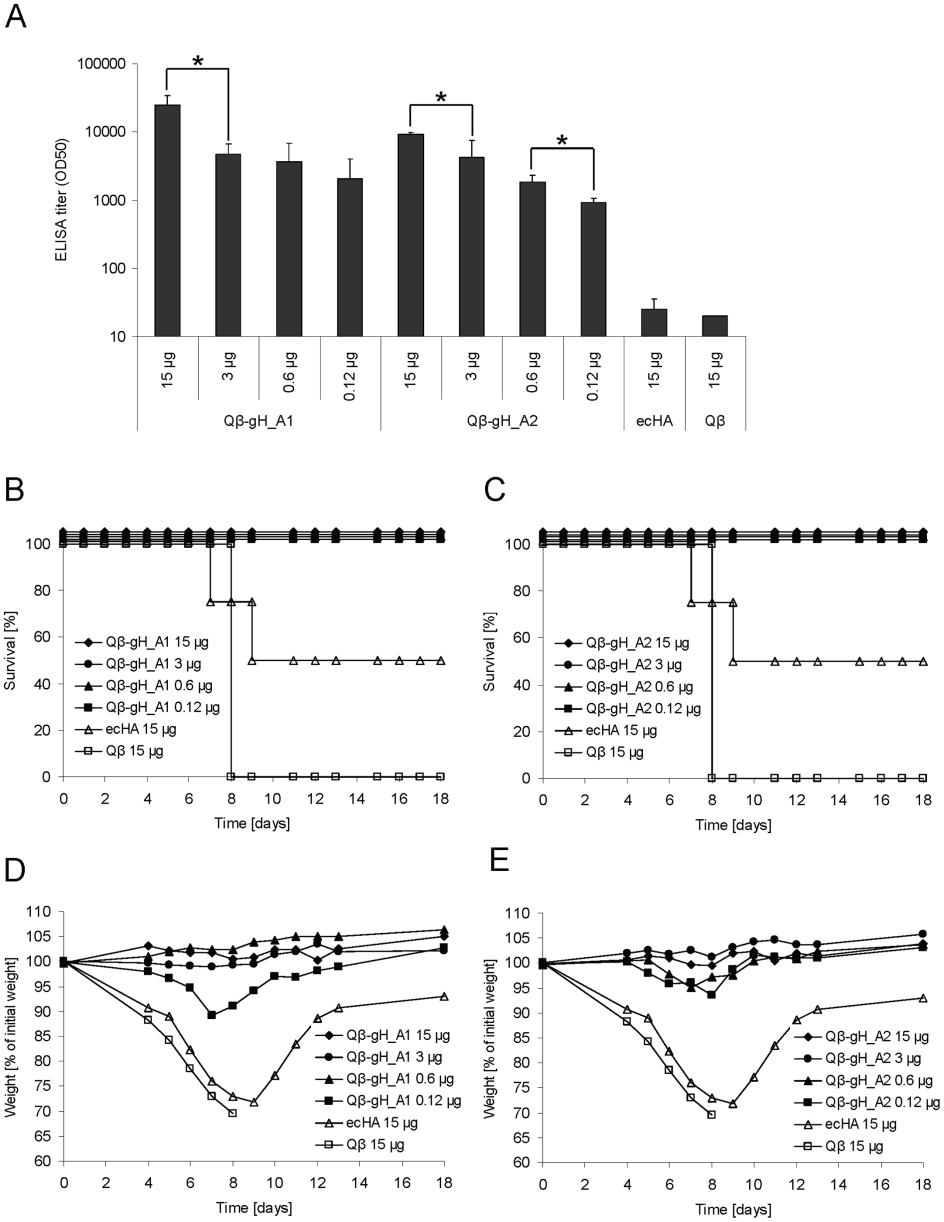
Titration of Qβ-gH_A1 and Qβ-gH_A2 vaccines of influenza A/PR/8/34. **A** Groups of six-weeks old female BALB/c mice (n = 4) were immunized subcutaneously once with 15, 3, 0.6 or 0.12 μg of Qβ -gH_A1 and Qβ -gH_A2 vaccines. IgG antibody titers specific for native HA protein of A/PR/8/34 were measured at day 18 after immunization. ELISA titers are indicated as IgG titer at which half-maximal OD was reached (OD50). Student's T-test was used to compare groups statistically. Significant differences between groups receiving different doses of vaccine are outlined with *, p<0.05. **B-E** On day 21 mice were challenged i.n. with 4× LD50 of m.a. influenza virus A/PR/8/34. Survival **B,C** and weight loss **D,E** of individual mice were monitored for 18 days post challenge.

Next, the dose response for the 2 most promising vaccines was established. Groups of BALB/c mice (n = 4) were immunized once subcutaneously with 15, 3, 0.6 or 0.12 μg of Qβ -gH_A1 and Qβ -gH_A2 vaccines and IgG titers against native ecHA were determined three weeks later. Please note that 15 µg of conjugate vaccines correspond to total amounts of conjugate vaccine and not amounts of globular domain protein contained in the vaccine. Figure 3A shows that immunization with as little as 0.12 μg of the conjugate vaccines induced higher antibody titers than immunization with 15 μg of native ecHA. To assess the protective potential of the vaccines, mice were challenged i.n. with influenza A/PR/8/34 virus and monitored for mortality (Figure 3B, 3C) and morbidity (Figure 3D, 3E). All mice immunized with conjugate vaccine were completely protected from lethal challenge and showed no signs of morbidity whereas mice immunized with native ecHA were only partially protected. As expected Qβ -immunized control mice succumbed rapidly to infection.

### *In vivo* efficacy of Qβ -gH vaccines against drifted Influenza strains

**Figure 4:**
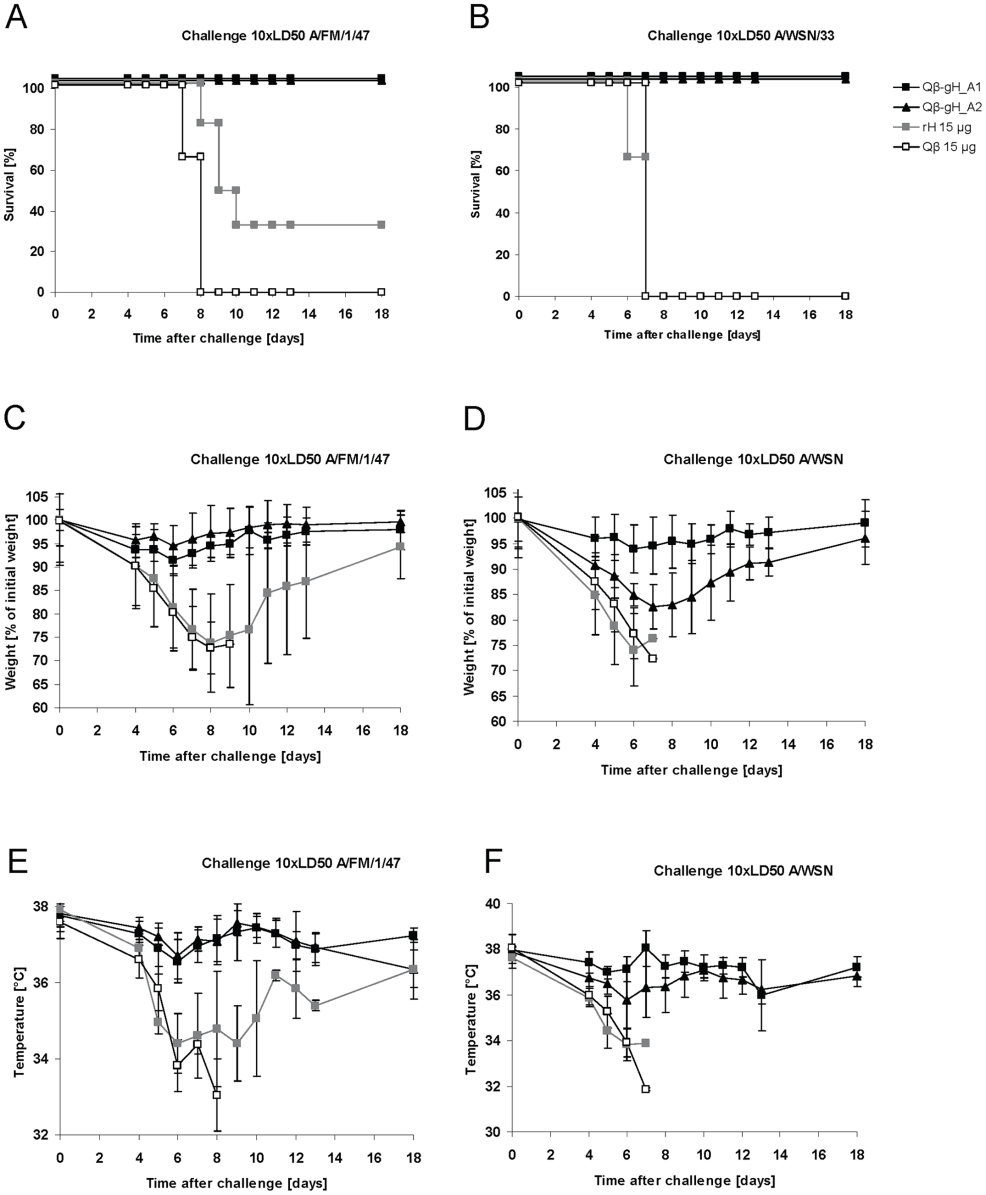
*In* vivo efficacy of Qβ -gH vaccines of influenza A/PR/8/34 against drifted influenza strains. **A-F** Groups of six-weeks old female BALB/c mice (n = 6) were immunized subcutaneously one with 15 μg of the Qβ -gH_A1 or Qβ -gH_A2 vaccine, 15 μg of the recombinant trimeric native HA protein (rH) or 15 μg Qβ. Three weeks after immunization mice were infected i.n. with a lethal dose (10× LD50) of influenza A/FM/1/47 (**A, C, E**) or A/WSN/33 (**B, D, F**). Survival **A, B**, weight loss **C, D** or temperature drop **E, F** of individual mice were monitored for 18 days post challenge.

Next induction of cross-protection against drifted H1N1 strains such as A/FM/1/47 and A/WSN/33 was assessed. A/FM/1/47 has 38 changes, A/WSN/33 has 36 changes in amino acids compared to A/PR/8/34 within the region of the globular domain protein. The number of amino acid changes within the antigenic sites is comparable between A/FM/1/47 and A/WSN/33, which is surprising, as the difference between A/WSN/33 and A/PR/8/34 is only one year compared to a difference of thirteen years between A/FM/1/47 and A/PR/8/34. Groups of BALB/c mice (n = 6) were immunized subcutaneously with 15 μg of the Qβ -gH_A1 or Qβ -gH_A2 vaccines, 15 μg of native ecHA or 15 μg Qβ. Three weeks after immunization mice were infected i.n. with a lethal dose (10× LD50) of mouse adapted influenza A/FM/1/47 or A/WSN/33 and animals were monitored for morbidity and mortality. As shown in Figure 4, control mice immunized with Qβ VLP all succumbed to infection. Mice vaccinated with ecHA protein, with a few exceptions, also succumbed to infection. In contrast, mice immunized with Qβ -gH_A1 or Qβ -gH_A2 vaccines showed almost no morbidity and all survived. This result is surprising as a vaccine composed of A/Weiss/43, A/PR/8/34 and B/Lee/40 completely failed to protect against an influenza epidemic of influenza A/FM/1/47 strain in humans in 1947, an incidence called “The total influenza vaccine failure of 1947” [24]. This vaccine failure was reproduced in mice using an inactivated virus for immunization [24], high-lighting the potency of our vaccines.

Since there was no difference in the efficacy between the two vaccine candidates we arbitrarily selected one candidate, Qβ -gH_A1, for further analysis.

### Enhanced protective antibody responses upon booster injections and in the presence of Alum

**Figure 5:**
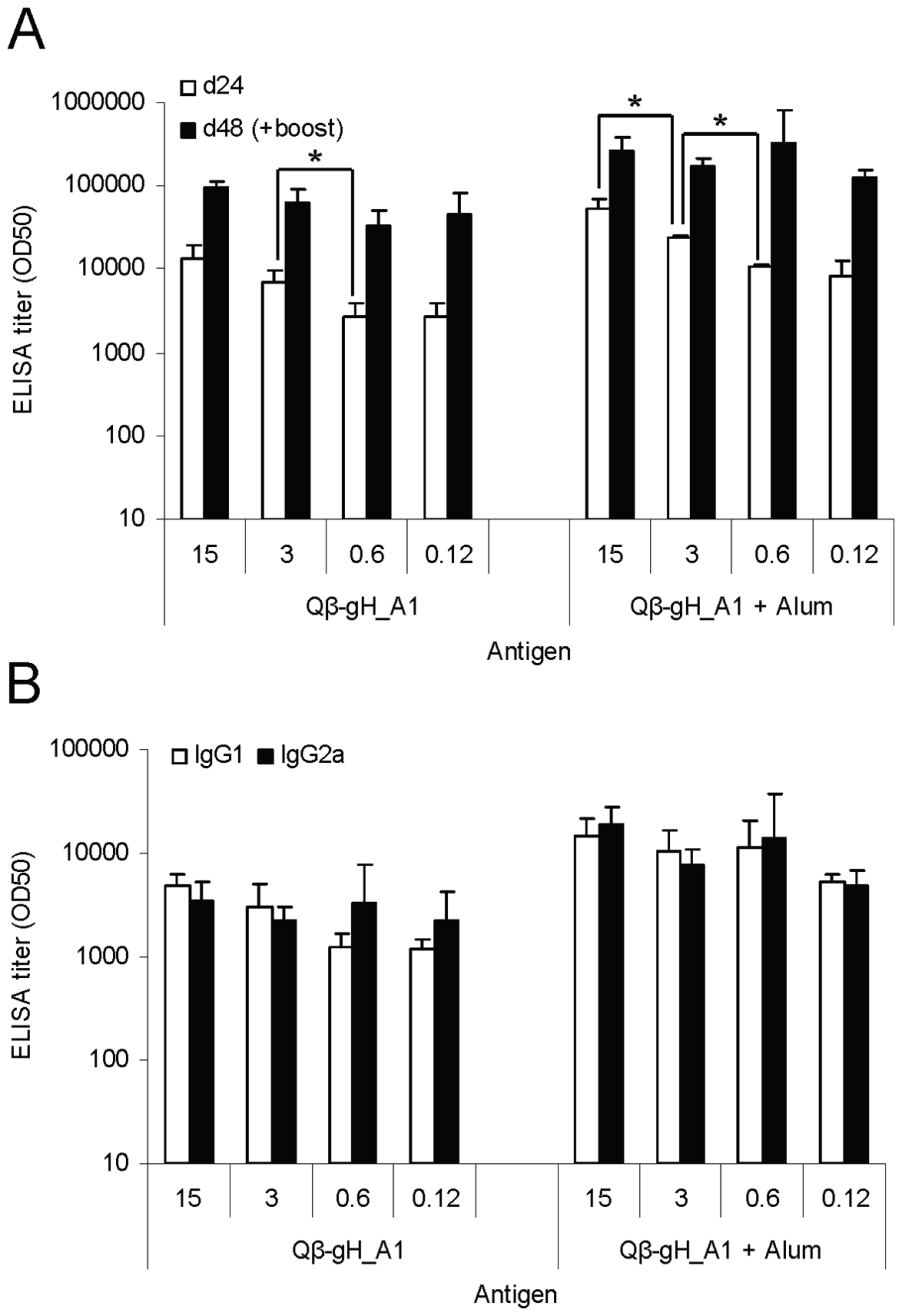
Boost and adjuvant effect on the efficacy of the Qβ-gH_A1 vaccine of influenza A/PR/8/34. **A** Groups of six-weeks old female BALB/c mice (n = 4) were immunized subcutaneously once or twice at day 0 and day 24 with or without Alum with 15, 3, 0.6 or 0.12 μg of the Qβ -gH_A1 vaccine. IgG antibody titers specific for native HA protein of A/PR/8/34 were measured at day 24 and day 48 after immunization. ELISA titers are indicated as IgG titer at which half-maximal OD was reached (OD50). Student's T-test was used to compare groups statistically. Significant differences between groups receiving different doses of vaccine are outlined with *, p<0.05. **B** IgG1 and IgG2a subtypes were measured at day 24 after immunizations. ELISA titers are indicated as IgG1 and IgG2a titers at which half-maximal OD was reached (OD50).

It is generally assumed that immunologically naive individuals need two injections of influenza vaccines to mount protective responses. Therefore we tested whether booster injections or addition of Alum would further enhance the IgG response to the conjugate vaccine. BALB/c mice (n = 4) were immunized subcutaneously once or twice with or without the addition of the adjuvant Alum with 15, 3, 0.6 or 0.12 μg of the Qβ -gH_A1 vaccine. IgG antibody titers against native ecHA were determined by ELISA 24 days after the first and second immunization. Irrespective of the amount of vaccine injected, a second immunization induced a significantly increased antibody responses and formulation in Alum further enhanced the response (Figure 5A). A significant dose response was seen after first immunization, with and without Alum, whereas after second immunization no significant difference between groups was observed. Furthermore, we could show that ELISA titres correlated with HAI titers (Table 2).

**Table 2:** Hemagglutination inhibition assay.

Antigen	Dose [μg]	HAI Titer d24	HAI Titer d48
Qβ-gH_A1	15	144	832
	3	112	608
	0.6	104	320
	0.12	128	160
Qβ-gH_A1/Alum	15	576	2944
	3	208	1664
	0.6	52	2496
	0.12	52	1160

Enhanced HAI titers upon booster injections and in the presence of Alum. Groups of BALB/c mice (n = 4) were immunized subcutaneously once or twice with or without the addition of the adjuvant Alum with 15, 3, 0.6 or 0.12 µg of the Qβ -gH_A1 vaccine (total amount of conjugate vaccine and not the amount of globular domain protein contained in the vaccine). HAI titers were determined 24 days after the first and second immunization.

Efficacy of influenza vaccination is often influenced by the antibody isotype induced by the vaccine. Production of IgG2a antibodies in mice has been associated with increased efficacy of influenza vaccination [25], an observation which might be explained by its superiority in activation of Fc receptor-mediated effector functions compared to IgG1 [18]. Subunit vaccines [26] as well as Alum adjuvanted vaccines [17] have a tendency to induce rather IgG1 than IgG2a antibody responses. As can be seen in Figure 5B, conjugation of the subunit protein gH_A1 to virus-like particles resulted in strong IgG2a responses even in the presence of Alum. This was in contrast to HA protein alone, which induced more IgG1 than IgG2a, with and without Alum (data not shown).

### Evaluation of the Qβ -gH vaccine technology for a currently circulating H1N1 influenza A strain

**Figure 6:**
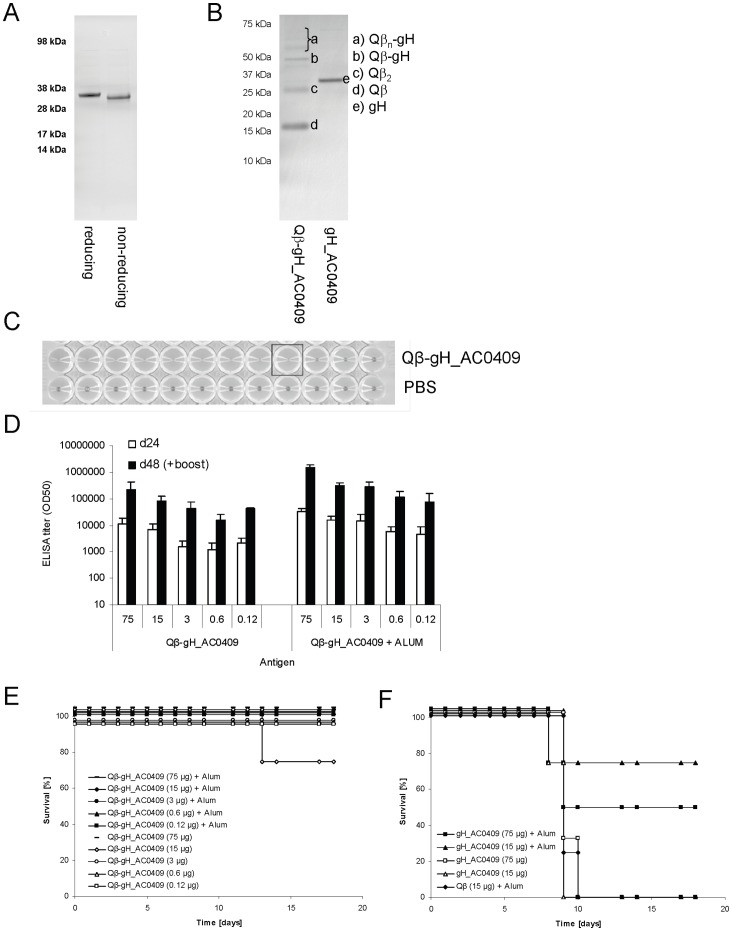
Evaluation of the Qβ -gH vaccine technology for currently circulating H1N1 strain namely influenza A/California/04/09 virus. **A** gH_AC0409 protein after purification and refolding was analyzed on a reducing or non-reducing denaturing Commassie stained SDS-PAGE gel. A shift of the bands between the two lanes can be seen. 4 µg of total protein were loaded in one lane. **B** Qβ -gH_AC0409 conjugate vaccine was analyzed on a reducing denaturing Commassie stained SDS-PAGE gel. 4 µg of total protein were loaded in one lane. **C** Assessment of the quality of Qβ -gH_AC0709 conjugate vaccine in a Hemagglutination assay. 5.5 pg of the vaccine were enough to agglutinate chicken red blood cells. **D** Groups of six-weeks old female BALB/c mice (n = 4) were immunized subcutaneously twice with titrating amounts of Qβ -gH_AC0409 vaccine with or without ALUM (90 µg of ALUM/mouse). IgG antibody titers specific for native HA protein of A/California/04/09 were measured at day 24 and day 48 after immunization. ELISA titers are indicated as IgG titer at which half-maximal OD was reached (OD50). **E, F** Twenty days after second immunization mice were infected i.n. with a lethal dose (4× LD50) of m.a. influenza A/PR/8/34 and survival of individual mice was monitored for 18 days.

We next expressed the globular domain of the currently circulating influenza A/California/04/09 in the same manner as gH_A1 of A/PR/8/34 and coupled the protein to Qβ. As expected from the disulfide bridges present in the protein, reducing the protein caused a shift of the band in an SDS page (Fig 6A). The protein was then conjugated to Qβ -VLPs (Figure 6B). The quality of the vaccine was assessed in a hemagglutination assay (Figure 6C). We quantified the minimal amount of Qβ -gH_AC0709 VLPs needed to agglutinate turkey RBCs and found that it was in a similar range as the minimal number of influenza virus particles (both normalized for the number of HA molecules/particle). To assess immunogenicity of the vaccine, groups of BALB/c mice (n = 4) were subcutaneously immunized twice with titrating amounts of Qβ -gH_AC0409 vaccine with or without Alum and antibody titers were measured by ELISA using native trimeric full-length HA from influenza A/California/04/09. High, specific antibody-titers could be induced which were further enhanced by the addition of Alum as through boosting (Figure 6D). Twenty days after the second immunization mice were infected with a lethal dose of mouse adapted influenza A/PR/8/34 virus (4× LD50) and morbidity and mortality was monitored. Influenza A/PR/8/34 was chosen because a mouse adapted influenza A/California/04/09 strain was not available and because we were interested to see whether mice were cross-protected against this heavily drifted strain. It is important to note, that there is only 70 percent amino acid sequence identity between the globular domain of A/PR/8/34 and the one of A/California/04/07. Nevertheless, mice immunized with Qβ -gH-AC0409 conjugate vaccine were well protected even when immunized with as little as 125 ng. In contrast, mice immunized with high doses of the globular protein alone adjuvanted with Alum were only partially protected, while control mice rapidly succumbed to the infection (Figure 6E, F). Overall this result showed that the Qβ -gH_AC0409 vaccine was highly immunogenic and had an excellent broad protective potential.

### Evaluation of the Qβ-gH vaccine technology for H1N1, H3N2, H5N1 influenza A and influenza B strains

Despite the fact that amino acid sequences of influenza HA proteins of different influenza strains differ considerably even within one subtype, the structure of the different influenza HA proteins of different strains is remarkably conserved. Hence, we reasoned that structural alignment of the prototype gH_A1 protein of A/PR/8/34 with other influenza HA proteins would allow the design of gH_A1 proteins of any influenza A or B strain. The selected strains were the components of the conventional trivalent influenza vaccine of 2008/2009 namely A/Brisbane/59/07 (H1N1), A/Uruguay/716/07 (H3N2) and B/Brisbane/3/07 (influenza B). Two prototype H5N1 strains also were selected: A/Vietnam/1203/04 and A/Indonesia/5/05. Through structural alignment, the globular domain proteins were designed and production was performed in the same manner as for the prototype globular domain protein of influenza A/PR/8/34. High yields of the individual proteins were obtained (Table 3). Groups of BALB/c mice (n = 5) were subcutaneously immunized once with 10 μg of the gH proteins in Alum. Serum samples were collected three weeks after immunization and IgG antibody titers against native trimeric full-length HA of the individual strains were determined. As can be seen in Table 3, high antibody titers against native proteins were induced. By way of comparison, the values for gH protein of A/California/04/09 are also indicated in the table. Thus, the herein described method to produce globular HA domains exhibiting the right structure is suitable for all influenza strains tested.

**Table 3:** Production and evaluation of gH proteins of different subtypes.

Subtype	Strain	Production yield per liter fermentation [g/l]	Immunogenicity of gH ELISA titer against rH [0D50]
Influenza A, H1N1	A/Brisbane/59/07	2.9	1′385
Influenza A, H3N2	A/Uruguay/716/07	0.7	1′033
Influenza B	B/Brisbane/3/07	2.1	1′255
Influenza A, H5N1, clade1	A/Vietnam/1203/04	2.3	3′714
Influenza A, H5N1, clade2 Influenza A, H1N1	A/Indonesia/5/05 A/Califonia/04/09	1.3 2.5	21′264 11′625

Evaluation of the gH vaccine technology for H1N1, H3N2, H5N1 influenza A and influenza B strains. gH proteins of the influenza strains which were the components of the conventional trivalent influenza vaccine of 2008/2009 namely A/Brisbane/59/07 (H1N1), A/Uruguay/716/07 (H3N2) and B/Brisbane/3/07 (influenza B) as well of two prototype H5N1 strains namely A/Vietnam/1203/04 and A/Indonesia/5/05 were produced and. Production yields of recombinant proteins after purification and refolding per liter fermentation are shown. Groups of BALB/c mice (n = 5) were subcutaneously immunized once with 10 µg of the gH proteins in Alum. IgG titers against native trimeric full-length HA of the individual strains were determined three weeks later.

## Discussion

The major limitations of current influenza vaccines are their slow production time-lines combined with low yields and limited immunogenicity. Here we describe a novel influenza vaccine technology that has the potential to overcome these limitations: The HA globular domain is produced in bacteria at very high yield and subsequently coupled to Qβ-VLPs which enhances their immunogenicity roughly 100-fold.

We set out to recombinantly produce the globular domain protein of influenza HA *in E.coli*. Upon screening of eleven HA1 fragments we identified several globular domain fragments of the influenza hemagglutinin protein of an H1N1 strain which could be readily expressed in *E. coli* and upon *in-vitro* refolding displayed a native structure as demonstrated by biochemical and immunological methods. Conjugation of this homogenous protein to Qβ VLP resulted in a highly immunogenic H1N1 influenza A vaccine against the prototype mouse strain A/PR/8/34. Through structural alignment of the prototype globular domain fragment with influenza HA proteins we selected from other influenza subtypes and even of influenza B strain, we designed and produced globular domain proteins of H1N1, H3N2, H5N1 and influenza B viruses. These proteins exhibited the authentic structure which enabled the induction of specific antibodies. This demonstrates our influenza vaccine technology can be generally applied for different influenza types and subtypes.

Antigens coupled to Qβ have been used for vaccination in numerous preclinical and clinical studies. Antigens coupled to Qβ are highly repetitive, strongly enhancing their immunogenicity for B cells. The RNA contained within Qβ, which is a ligand for TLR7/8, further enhances the immunogenicity of the antigens and drives IgG responses towards the more protective IgG2a isotypes [27], [28], [29]. In addition, strong Th1 as well as cytotoxic T cell responses against the attached antigen are also induced [27], [30] (see also the accompanying paper). It remains therefore possible that induction of HA-specific T cell responses contributes to protection. However, it is likely that antibodies are the most important effector mechanism for efficacy, a notion that is supported by the finding that the construct gH_B3, which induced the weakest antibody response, also was least efficient at protecting mice against lethal infection (Fig 2).

In the mouse, antibody responses against antigens coupled to Qβ are typically enhanced 10–100 fold compared to immunization with free antigen (Number of antigens tested: >30). In humans, after a single injection, a response rate of 100% has been observed in the approximately 300 subjects immunized to date [31], [32], [33], [34], [35]. Furthermore, rapid boosting regimens (e.g. weekly or biweekly) could bring antibody titers to very high levels within a few weeks. These immunological properties could be of key significance for potential outbreaks of H5- or H7- based viruses, where the population is essentially immunologically naïve and conventional vaccines suffer from low immunogenicity, requiring booster injections to reach protective levels.

The here described VLP-based technology is a modular approach which allows separate synthesis of the native VLP and target antigen. This has the advantage that the size and structure of the recombinant target antigen is not limited by the folding constraints of the VLP monomer and the subsequent self-assembly process. [16]. The modular approach has the advantage that the carrier Qβ VLP can be produced in large amounts independently of the production of gH proteins by recombinant expression in *E. coli*. well ahead of the influenza season. After WHO selection of the influenza strain, which should be contained in the vaccine, the gene of interest can be synthesized and cloned into an expression vector and the protein of interest is expressed as inclusion bodies. Inclusion bodies can be easily purified to high purity which makes extensive purification of the protein after *in-vitro* refolding dispensable. Conjugation of gH proteins to Qβ VLPs is done in an efficient and well controlled *in-vitro* step. Compared to current manufacturing systems of influenza vaccines this approach has the advantage that it does not depend on the production of a high growth seed virus which in some instances can be cumbersome. Recombinant production also is more safe as the risk of incomplete inactivation of influenza virus is omitted. Quantification of the gH protein can be done by standard protein quantification methods and therefore is independent of reagents needed to do SRID assay which is used for quantification of HA protein in current manufacturing. These reagents have to be updated every year and delays in delivery of these reagents are quite common.

Current conventional influenza vaccines rely on achieving a good match between circulating strains and the isolates included in the vaccine [36], but such a match is often difficult to attain despite the huge efforts of the WHO to select the best matching strains [37] Our prototype influenza vaccine Qβ -gH_A/PR/8/34 showed an unexpected ability to cross-protect against highly drifted strains. One hypothesis to explain this phenomenon is that bacterially expressed HA antigen is devoid of glycosylation and hence conserved epitopes which normally are hidden through glycosylation might appear and induce some cross-protective antibody responses [35].

Taken together, the here described recombinant influenza technology allows the fast and easy production of large amounts of vaccines based on the influenza HA globular domain coupled to VLPs. This technology may be used to produce seasonal influenza vaccines as well as vaccines against newly emerging pandemic influenza outbreaks.
